# Uncommon Blepharitis

**DOI:** 10.3390/jcm13030710

**Published:** 2024-01-25

**Authors:** Antonio Di Zazzo, Giuseppe Giannaccare, Edoardo Villani, Stefano Barabino

**Affiliations:** 1 Ophthalmology Operative Complex Unit, University Campus Bio-Medico, 00128 Rome, Italy; 2 Eye Clinic, Department of Surgical Sciences, University of Cagliari, 09124 Cagliari, Italy; 3 Eye Clinic, San Giuseppe Hospital, IRCCS Multimedica, University of Milan, 20123 Milan, Italy; 4 Ocular Surface & Dry Eye Center, Department of Ophthalmology, ASST Fatebenefratelli SACCO, Università di Milano, 20157 Milan, Italy

**Keywords:** blepharitis, anterior blepharitis, posterior blepharitis, meibomian gland dysfunction, blepharitis treatment, bortezomib, cetuximab, TNF-α inhibitors, dupilumab

## Abstract

Blepharitis is a common chronic inflammatory condition affecting the eyelid margins; the pathophysiology of blepharitis is complex and not fully understood. The disease is anatomically divided into anterior (inflammation of eyelashes) and posterior (meibomian gland dysfunction) types. Diagnosis relies on clinical examination, revealing characteristic features like scurf, vascular changes, and meibomian gland dysfunction. The main goals of blepharitis treatment are symptom relief, recurrence prevention, and complication risk minimization. Treatment options include lid hygiene, topical and systemic antibiotics, topical corticosteroids, and omega-3 supplements. However, it is important to highlight reported cases of blepharitis as side effects of systemic therapies, particularly in the context of chemotherapy, bortezomib, cetuximab, TNFα inhibitors, and dupilumab. It is crucial to monitor patients undergoing such treatments regularly and attentively in order to promptly set up adequate supportive therapy. Of even more importance is future research on the pathophysiological mechanisms responsible for the occurrence of these ocular side effects in order to find a nosological cure for the issue.

## 1. Introduction

Blepharitis is a common chronic inflammatory condition that affects the eyelid margins of any age group [1,2]. It can be linked to various systemic conditions, especially rosacea and seborrheic dermatitis [3]. Additionally, it is associated with other eye-related conditions such as dry eye, chalazion, conjunctivitis, and keratitis [4].

The pathophysiology of blepharitis is complex and not fully understood. It is believed to be a multifactorial disease with various underlying causes. It can be anatomically subdivided into anterior and posterior blepharitis, even if considerable overlap exists between the two types [5].

**Figure 1:**
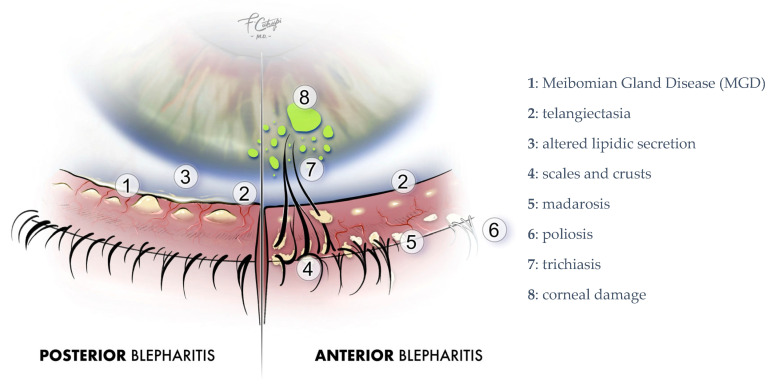
Blepharitis clinical signs. Image courtesy of Dr. Francesco Cutrupi.

Anterior blepharitis, which is the inflammation of the eyelashes and follicles, is usually infectious, with Staphylococcus Aureus being the most common organism responsible. The bacteria can colonize the skin and eyelid margins, leading to inflammation and damaging the eyelid structures. The process through which bacteria induce the symptoms of blepharitis is not completely comprehended. It might involve direct irritation caused by bacterial toxins and/or an increased immune response against *S. aureus* [6]. Anterior blepharitis is also associated with seborrheic dermatitis [7]. Figure 1 shows the clinical characteristics of a typical patient with blepharitis.

Posterior blepharitis, on the other hand, is caused by dysfunction of the meibomian glands, which secrete lipids that form the outer layer of the tear film. Dysfunction of the meibomian glands can lead to alterations in the lipid composition of the tear film, resulting in evaporative dry eye and inflammation of the eyelid margins [8,9].

The diagnosis of blepharitis is primarily clinical and based on the patient’s symptoms and physical examination findings [10]. Blepharitis is often accompanied by a range of symptoms, including a burning sensation, irritation, tearing, photophobia, blurred vision, and red eyes. These symptoms are typically more severe in the morning because during sleep, the inflamed eyelids come into closer contact with the surface of the eye. In addition, during the night, tear production decreases, which, in association with the constant release of inflammatory mediators, contributes to corneal damage [4,10].

Clinical examination has revealed several characteristic features of blepharitis, including scurf, telangiectatic vascular changes of the eyelid margin, inspissated meibomian glands, conjunctival hyperemia, punctuate keratopathy, corneal vascularization, and ulceration. Hard crusts around the base of the eyelashes, known as collarets, are typical of staphylococcal blepharitis. Seborrheic blepharitis is characterized by hyperemia, lipid secretion, and soft crusts at the lid margin and eyelashes. Patients with longstanding chronic blepharitis may develop hypertrophy of the lid margin, scars, madarosis, trichiasis, and poliosis. Posterior blepharitis typically has more pronounced symptoms but fewer clinical signs, with meibomian gland orifices exhibiting small drops of lipid secretion and pressure on the tarsus releasing a thick lipid secretion [4,10].

The treatment of blepharitis depends on the underlying cause of the disease. The main goals of treatment are to alleviate symptoms, prevent recurrences, and minimize the risk of complications. Treatment options include:

- Lid hygiene: Regular lid hygiene, including warm compresses and lid scrubs, can help remove debris and bacteria from the eyelid margins and improve meibomian gland function [2].
- Topical antibiotics: In cases of bacterial infection, topical antibiotics such as erythromycin or bacitracin may be prescribed.
- Topical corticosteroids: Topical corticosteroids can be used to reduce inflammation in cases of severe blepharitis [11].
- Systemic antibiotics: In cases of severe or chronic blepharitis, systemic antibiotics such as doxycycline or azithromycin may be prescribed [12].
- Omega-3 supplements: Omega-3 supplements have been shown to improve meibomian gland function and reduce inflammation in patients with posterior blepharitis [13].

However, several cases of blepharitis have been reported in the literature as side effects of various systemic therapies in settings that we could define as “extreme”.

## 2. Blepharitis and Chemotherapies

Several types of chemotherapy agents have been associated with the development of blepharitis, including taxanes and antimetabolites. Each of these drugs can cause blepharitis through different mechanisms.

Taxanes, such as paclitaxel and docetaxel, induce blepharitis [14], likely due to direct damage to the meibomian glands that leads to the disruption of the tear film, causing dry eyes and subsequent blepharitis [15].

Antimetabolites, such as 5-fluorouracil (5-FU) and methotrexate, can cause blepharitis through a variety of mechanisms. These agents can directly damage the meibomian glands, leading to a decrease in tear film quality and subsequent blepharitis. Additionally, 5-FU has been shown to induce an inflammatory response in the eyelids, leading to redness, swelling, and irritation [16,17,18].

Additionally, chemotherapy can compromise the immune system, allowing for the overgrowth of bacteria on the eyelids, further contributing to blepharitis.

Systemic chemotherapy-induced blepharitis can present with a variety of the classic symptoms; however, in severe cases, the eyelids may become so inflamed that the patient is unable to fully close their eyes, causing corneal exposure and potential damage to the cornea [16,17,18].

The management of systemic chemotherapy-induced blepharitis should be tailored to the individual patient and the severity of their symptoms.

## 3. Blepharitis and Bortezomib

Bortezomib is a proteasome inhibitor used in the treatment of multiple myeloma that has been associated with the development of blepharitis. Bortezomib-induced blepharitis is characterized by the presence of crusty debris along the eyelid margins, redness, and irritation [19].

The exact mechanism of bortezomib-induced blepharitis is not well understood, but it is thought to be related to the drug’s effect on the meibomian glands. There is a hypothesis that bortezomib could hinder the ubiquitin pathway, resulting in the accumulation of proapoptotic molecules in the meibomian gland. This, in turn, could cause blepharitis and recurrent chalazia as secondary effects [19,20]. Simultaneously, bortezomib is known to affect other inflammatory pathways such as NF-kB, JAK/STAT, and MAP kinase, which can lead to the release of pro-inflammatory cytokines. The interference of all these pathways in the eyelids can contribute to inflammatory flares that cause blepharitis and/or chalazia [21,22].

Management of bortezomib-induced blepharitis is challenging, and treatment options are limited. Conservative measures, such as warm compresses and lid hygiene, are often recommended to manage symptoms. Topical antibiotics and corticosteroids may also be used to control inflammation and prevent secondary infections. If eyelid complications persist after this treatment, omission of bortezomib should be considered with a switch to alternative proteasome inhibitors (carfilzomib or ixazomib). Oral doxycycline can be added if no improvement is shown [20,21,22].

## 4. Blepharitis and Cetuximab

Cetuximab is a monoclonal antibody used in the treatment of metastatic colorectal cancer and head and neck cancer. While it is an effective therapy for these conditions, it has been associated with ocular side effects, including blepharitis [23]. While skin toxicity is a common side effect, ocular toxicity, including blepharitis, is much less frequent but still present [24].

The mechanism by which cetuximab causes blepharitis is not fully understood, but it is thought to be related to the drug’s effects on the epidermal growth factor receptor (EGFR) signaling pathway. Cetuximab binds to the extracellular domain of EGFR, preventing ligand binding and downstream signaling. This inhibition of EGFR signaling may disrupt the normal growth and differentiation of the eyelid epithelium, leading to blepharitis [25]. Furthermore, it has been suggested that cetuximab may target the cells in the meibomian glands that express EGFR, potentially leading to an alteration in their secretory function [26].

Management of cetuximab-induced blepharitis may involve conservative measures, such as warm compresses and lid hygiene, as well as topical antibiotics and corticosteroids to control inflammation and prevent secondary infections. In some cases, discontinuation of the drug may be necessary [27].

## 5. Blepharitis and TNF-α-Inhibitors

Tumor necrosis factor alpha (TNF-α) inhibitors are commonly used in the treatment of several autoimmune disorders such as rheumatoid arthritis, psoriasis, and Crohn’s disease [28]. Although they are effective in controlling inflammation, TNF-α inhibitors have been associated with various ocular side effects, including blepharitis [29,30].

Different TNF-α inhibitors have been reported to cause blepharitis, like infliximab, a chimeric monoclonal antibody, or adalimumab, a fully human monoclonal antibody. They both act by binding to TNF-α, thus inhibiting its action and reducing inflammation. The exact mechanism by which TNF-α inhibitors cause blepharitis remains unclear, but it has been suggested that it may be a paradoxical reaction thought to be secondary to genetic susceptibility, dominance of helper T cells types 1 and 17, and excessive immune response from interferon-α overproduction by plasmacytoid dendritic cells [29].

Management of TNF-α inhibitor-induced blepharitis is based on the usual conservative and preventive measures outlined in the previous paragraphs. The discontinuation of TNF-α inhibitors was associated with the improvement of blepharitis that were refractory to antibiotics and steroids, and it may be necessary.

## 6. Blepharitis and Dupilumab

Dupilumab is a monoclonal antibody used in the treatment of atopic dermatitis, asthma, and chronic rhinosinusitis with nasal polyposis [31,32,33]. While it is an effective therapy for these conditions, dupilumab has been linked to two forms of ocular surface disease: milder, non-specific conjunctivitis and keratitis that result in dry eyes, and more specific dupilumab-induced follicular conjunctivitis and limbitis [34].

Thanks to several prospective epidemiological studies, we know that approximately 28% of patients treated with dupilumab develop DIOSD (dupilumab-induced ocular surface disease). The median age was 38 years, with an equal male-to-female ratio. The average time to onset of ocular symptoms after starting dupilumab was 9.2 weeks. Most patients presented with bilateral conjunctival inflammation and blepharitis [35,36].

The mechanism by which dupilumab causes blepharitis is not fully understood, but it has been suggested that it may be related to the drug’s effects on the immune system and the ocular surface.

Dupilumab inhibits the signaling of both IL-4 and IL-13, which are cytokines involved in Th2-mediated inflammation. This may lead to an upregulation of Th1 response and IFN-γ-mediated inflammation. IFN-γ is a pro-inflammatory cytokine that has been established as a biomarker for evaporative dry eye disease and cell-mediated immune response in the mucosal immune compartment. As a result, a range of dupilumab-induced inflammatory conditions can occur, including blepharitis, tear film instability, meibomian gland dysfunction, goblet cell deficiency, exposure keratopathy, follicular conjunctivitis and limbitis, punctual stenosis, and periocular dermatitis [37,38].

The management of these ocular surface side effects is determined by the degree of inflammation and can range from the use of local steroids (such as fluorometholone 1%, dexamethasone 0.1%, and prednisolone) to the combination of tacrolimus eye ointment 0.03% or cyclosporine eye drops. In rare instances, ocular inflammation does not improve with any eye drops, and the discontinuation of dupilumab therapy is necessary [34,39].

## 7. Blepharitis and Epidermal Growth Factor Receptor (EGFR) Tyrosine Kinase Inhibitors

The identification of EGFR as an oncogene has led to the development of anticancer therapeutics directed against EGFR (called “EGFR inhibitors”, EGFRi), including gefitinib [40], erlotinib [41], afatinib, brigatinib, and icotinib [42,43] for lung cancer, and cetuximab for colon cancer. However, sight-threatening ocular adverse effects, like corneal ulcers and perforation, can occur due to the expression of EGFR on limbal and conjunctival epithelia [44]. This therapy is often associated with severe trichiasis (misdirection of lashes), trichomegaly (thickening of lashes), and hypertrichosis (increased number of lashes and brow hairs) [45]. Multiple reports have also described patients being treated for colorectal cancer with cetuximab presenting with severe squamous blepharitis [26].

Epidermal growth factor receptor is a transmembrane glycoprotein in the tyrosine kinase growth factor receptor family. It is constitutively expressed in many normal epithelial tissues, including the skin, glands (including the lacrimal gland), hair follicles, conjunctiva, and cornea [46]. More specifically, EGFR is expressed in the basal epithelial cells of limbal and conjunctival epithelia and throughout the corneal epithelium. Epidermal growth factor binds to these cell surface receptors and acts as an important regulator of normal cell proliferation and survival. Overexpression of growth factor receptors, including EGFR, is a fundamental element contributing to the growth and progression of many solid tumors. This can lead to increased or uncontrolled proliferation, decreased apoptosis, enhanced tumor cell motility, and angiogenesis [47].

The management of toxicity from EGFR inhibitors involves the use of artificial tears for the treatment of related dry eyes; trichiasis must be treated with the removal of excess eyelashes by an ophthalmologist, for blepharitis, eyelid hygiene and topical cortisone are recommended, and in cases of severe blepharitis, doxycycline 50 mg twice daily for 2 weeks, followed by 50 mg once daily for 4 weeks [47]. 

Table 1 presents a summary of the essential information regarding blepharitis cases induced by the systemic treatments discussed in this article.

## 8. Discussion

The increasing number of alternative treatments for several severe autoimmune and tumoral diseases has improved the quality of life of patients with these diseases. Side effects and complications related to long-lasting chronic diseases and medications, as well as improved survivorships, have led patients to focus more on symptoms and signs which may limit their normal daily activities by modifying aesthetics and quality of vision [48]. Therefore, uncommon blepharitis, mainly related to systemic and topical treatments of such novel chronic diseases, should be adequately managed by ophthalmologists, since a high number of patients will be affected by such conditions. Tear film instability is related mainly to a loss of functional visual acuity in activities which require a high level of focus, such as driving, working, and reading, as well as persistent ocular discomfort that can reach the level of severe burning, dryness, and pain, which may limit the lives of such people. Patients will continuously return to ophthalmologists for an often misdiagnosed and untreated issue, without any improvement of their condition. They will not feel understood, potentially that they are annoying the doctor, and then frustrated. As such, local and potentially not severe complications will worsen to become a large, persistent psychological condition, which can be very difficult to solve.

The ophthalmologist’s awareness of such uncommon blepharitis should also be complemented by the clear need for prophylactic management. Patients should be pre-informed before starting therapy of uncommon but possible issues, and advised to promptly start daily warm compresses with lid hygiene, as well as administering, with a strict schedule, muco-mimetic or amphipathic lubricating regenerating eye drops to allow the ocular surface system to react better to medicamentous toxic insults [49].

In rare cases, such prophylactics and therapeutic regimens may not be enough, and further customized local, mostly immunomodulating, medications are required. The critical goal of such management is to improve patients’ quality of life without interrupting the required systemic treatment; therefore, in these rare cases, the choice of drug should be driven by the chronicity more than the severity of the clinical picture.

The improved management of such less impactful compilations will definitively increase patients’ compliance with the actual, more significant, systemic treatments.

## 9. Conclusions

Blepharitis is a prevalent, long-term inflammatory disorder that impacts the margins of the eyelids across all age groups. Blepharitis is caused by both infectious and noninfectious factors, and it is commonly associated with several systemic conditions, notably rosacea and seborrheic dermatitis.

However, as we have observed, blepharitis can also be a consequence of different systemic medications, sometimes in such aggressive forms as to require their suspension. As a result, it is crucial to monitor patients undergoing such treatments regularly and attentively in order to promptly set up adequate supportive therapy.

For the same reason, it is essential to deepen our knowledge of the pathophysiological mechanisms responsible for the occurrence of these ocular side effects, to date still poorly understood. This will enable the development of more effective etiological therapies in the future.

## Figures and Tables

**Table 1:** Summary of the relationship between drugs and blepharitis.

Drugs	Symptoms	Mechanism	Therapy
Chemotherapeutics	Redness, inflammation, pain, swelling, itching, burning	Direct damage to the meibomian glands—impairment of the immune system resulting in bacterial proliferation	Customized therapy + eyelid hygiene ± antibiotics ± Cortisonics
Bortezonib	Redness, inflammation, pain, swelling, itching, burning	Not well understood—hypothesized to impair the ubiquitin pathway, resulting in accumulation of proapoptotic molecules in the meibomian gland—known to influence other inflammatory pathways such as NF-kB, JAK/STAT, and MAP kinase, which may lead to release of pro-inflammatory cytokines	Eyelid hygiene + topical antibiotics ± cortisonics—if the patient is refractory to therapy, either oral doxycycline or a switch to another molecule for therapy are recommended
Cetuximab	Redness, inflammation, pain, swelling, itching, burning	Not well understood—a paradoxical reaction is hypothesized due to a genetic predisposition	Eyelid hygiene + cortisonics + therapeutic wash-out ± topical antibiotics
TNF-α inhibitors	Redness, inflammation, pain, swelling, itching, burning	Not well understood—it is hypothesized that the inhibition of EGFR may disrupt the normal regeneration of the eyelid epithelium and that its interaction with the cells of the meibomian glands may alter their secretion.	Temporary suspension of therapy
Dupilumab	Redness, inflammation, pain, swelling, itching, burning	Not well understood—it is hypothesized that EGFR inhibition may alter the normal regeneration of the eyelid epithelium and that binding to meibomian gland cells may alter their secretion	Local steroids + tacrolimus or cyclosporine ± therapeutic wash-out

## Data Availability

The data presented in this study are available on request from the corresponding authors.
